# Enzootic Transmission of Yellow Fever Virus, Venezuela

**DOI:** 10.3201/eid2101.140814

**Published:** 2015-01

**Authors:** Albert J. Auguste, Philippe Lemey, Nicholas A. Bergren, Dileyvic Giambalvo, Maria Moncada, Dulce Morón, Rosa Hernandez, Juan-Carlos Navarro, Scott C. Weaver

**Affiliations:** University of Texas Medical Branch, Galveston, Texas, USA (A.J. Auguste, N.A. Bergren, S.C. Weaver);; Rega Institute, Leuven, Belgium (P. Lemey);; Ciudad Universitaria, Caracas, Venezuela (D. Giambalvo, M. Moncada, D. Morón, R. Hernandez);; Universidad Central de Venezuela, Caracas (J.-C. Navarro)

**Keywords:** Flavivirus, yellow fever virus, enzootic maintenance, Venezuela, phylogenetic analysis, phylogeography, coalescent analysis, evolution, viruses, vector-borne infections

## Abstract

Phylogenetic analysis of yellow fever virus (YFV) strains isolated from Venezuela strongly supports YFV maintenance in situ in Venezuela, with evidence of regionally independent evolution within the country. However, there is considerable YFV movement from Brazil to Venezuela and between Trinidad and Venezuela.

Yellow fever virus (YFV) is the prototype species for the genus *Flavivirus*. Historically, YFV is one of the most important human arboviral pathogens. It continues to cause large sporadic epidemics in Africa but typically emerges as epizootics among nonhuman primates in South America with or without associated human cases (*1*–*5*). YFV emergence is cyclical; outbreaks occur ≈7–10 years apart. Several phylogenetic studies have shown that YFV is locally maintained during these interepizootic periods in Peru (*6*), Brazil (*7*), and Trinidad (*4*). These studies also have indicated that the virus undergoes regionally independent evolution within some countries (*6*).

YFV has caused sporadic outbreaks in Venezuela; the most recently documented epizootic/epidemic occurred in 2005 (*8*). Although endemic to Venezuela, YFV has very rarely been isolated and characterized, and partial sequences have been determined only for 4 strains. Venezuela is located between Trinidad and Brazil, which have contributed major evidence for the enzootic maintenance of YFV in South America. Thus, sequencing Venezuelan YFV strains over a wide geographic area and temporal distribution might be valuable to test the hypothesis of local maintenance in Venezuela and to determine whether the virus moves regularly between Trinidad and Venezuela or between Venezuela and Brazil. Understanding the maintenance and spread of YFV in South America also is critical for developing effective surveillance and prevention strategies. We sequenced a prM/E gene fragment of 10 YFV isolates from 4 locations within Venezuela, spanning 6 years (2004–2010; Table 1). Additionally, we sequenced complete genomes for 5 representative isolates for comparison with 12 previously determined genomic sequences (*4*,*9*).

**Table 1:** Yellow fever virus strains sequenced in the study and their metadata, Venezuela*

Isolate ID	Source	Location	Year of collection	Passage history	GenBank accession no.
1A	Red howler monkey	Monagas	2004	Vero 2	KM388819
2A†	Red howler monkey	Guárico	2004	Vero 2	KM388817
3A	Red howler monkey	Portuguesa	2005	Vero 2	KM388820
4A	Red howler monkey	Portuguesa	2005	Vero 2	KM388821
5A	Human	Portuguesa	2005	Vero 2	KM388822
6A†	Human	Portuguesa	2005	Vero 2	KM388814
7A	Human	Portuguesa	2005	Vero 2	KM388823
8A†	Red howler monkey	Barinas	2006	Vero 2	KM388818
9A†	Red howler monkey	Apure	2007	Vero 2	KM388815
10A†	Red howler monkey	Monagas	2010	Vero 2	KM388816

*ID, identification. Red howler monkey, *Alouatta seniculus *species. †Complete genome sequences determined.

## The Study

The sporadic emergence of YFV in the Americas has been strongly associated with infection of red howler monkeys (*Alouatta seniculus*), which are particularly susceptible to disease. As exemplified in this study, nonhuman primate surveillance targeting this species remains an efficient strategy for monitoring enzootic YFV activity. Isolates made during a surveillance study aimed at investigating the ecology of infectious diseases in Venezuelan nonhuman primates were detected by cell culture and passaged once in Vero cells before sequencing, as previously described (*4*). Sequences were manually aligned in Se-Al (http://tree.bio.ed.ac.uk/software/seal/) and confirmed as nonrecombinant by using Recombination Detection Program (RDP4) (*10*). We obtained phylodynamic and phylogeographic estimates using Bayesian inference as implemented in BEAST v1.8.0 (*11*,*12*). We assessed the extent of geographic structuring using Bayesian tip-significance testing (*13*) based on the Markov chain Monte Carlo phylogenies estimated in BEAST. A Bayesian phylogeny was also inferred in Mr. Bayes (*14*) by using the General Time Reversible (GTR+I+Γ_4_) model for the complete open reading frame sequences.

**Figure 1:**
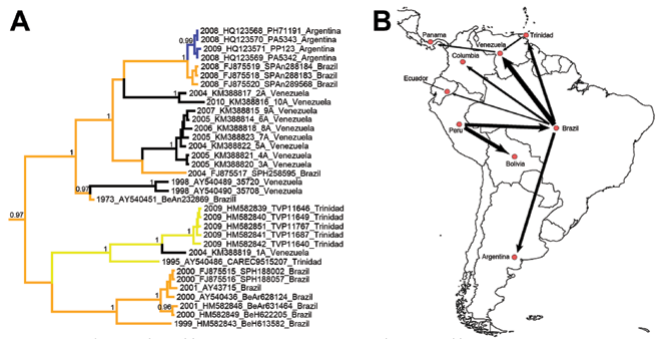
A) Bayesian maximum clade credibility (MCC) tree for YFV in the Americas based on 654 nt of the prM/E fragment. Taxon labels include year of isolation, GenBank accession number, strain designation, and country of isolation. Terminal branches of the tree are colored according to the sampled location of the taxon at the tip. Internal branches are colored according to the most probable (modal) location of their parental nodes. Nodes with posterior probabilities (clade credibilities) >0.95 are labeled accordingly in black. Scale bar indicates time in years. B) Magnified inset of the MCC phylogeny showing the tree topology for a subset of South American genotype I strains. C) Bayes factor (BF) test for significant non-zero rates indicating the statistical support for epidemiologically linked countries. Rates supported by a BF >5 are shown. The thickness of the arrows represents the relative strength by which the rates are supported. The Technical Appendix presents the details of the 124 sequences used in this study

Results of Bayesian tip-significance testing showed statistically significant geographic clustering among Venezuelan YFV strains. The association index, parsimony score, and maximum monophyletic clade statistics provided strong support (p<0.01) that strains from Venezuela cluster by location, suggesting that YFV is maintained for long periods within Venezuela. Similar results have been shown for Peru (*6*), Brazil, and Trinidad (*4*). The high posterior probabilities, >0.99, observed at all nodes that delineate Venezuelan strains further support these conclusions (Figure 1).

**Figure 2:**
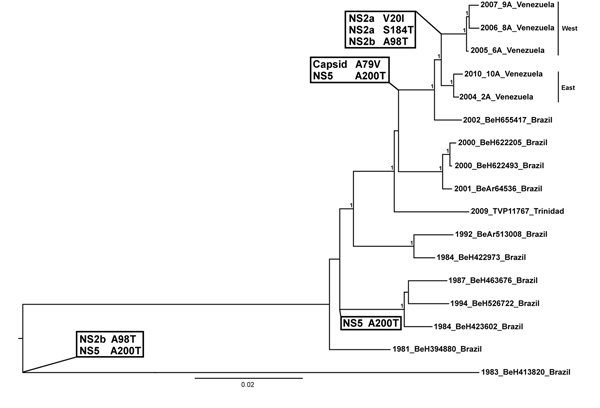
Midpoint rooted Bayesian Markov chain Monte Carlo phylogeny based on yellow fever virus (YFV) complete open reading frame sequences. Numbers at nodes indicate posterior probabilities >0.9. Eastern and western Venezuelan sequences are indicated. Substitutions resulting from nonsynonymous, synapomorphic mutations that define sequences in a clade/lineage are highlighted at relevant nodes. Two substitutions (NS2b A98T and NS5 A200T) occurred in earlier isolates from Brazil. The capsid A79V and NS5 A200T substitutions include the Brazilian isolate BeH655417, which lies directly basal to the Venezuela isolates. This indicates that these substitutions were probably present in the YFV progenitor when it was introduced into Venezuela. Furthermore, substitutions NS2a V10I, NS2a S184T, and NS2b A98T all appear to have arisen after YFV was introduced to Venezuela, further supporting enzootic YFV maintenance there. Taxon/tip labels include year of isolation, strain name and country where the virus was isolated. Scale bar indicates percentage of nucleotide sequence divergence.

We also found evidence of regionally independent evolution within Venezuela, as indicated by the existence of 2 phylogenetically distinct Venezuelan clades with posterior probabilities >0.99 (Figures 1, 2). The clade containing a 2004 strain (2A) from Guárico and a 2010 strain (10A) from Monagas represented all but one of the sequences from eastern Venezuela (i.e., east of Caracas; Table 1). The 7 other strains were collected on the western side of Venezuela (including Portuguesa, Apure, and Barinas States) (Table 1). Two YFV strains were collected in 2004 from eastern and western Venezuela. Despite their nearly synchronous collections in 2004, these sequences fell into distinct clades in the maximum clade credibility (MCC) phylogeny, indicating population subdivision. Although we cannot rule out sampling bias, these data suggest in situ evolution of YFV in Venezuela and regionally independent evolution in distinct geographic foci within the country. The mechanism promoting this population subdivision among YFV strains is unclear and requires further investigation. Further studies on the ecology of the areas where these viruses were isolated might help explain the observed population subdivision.

Although YFV had been maintained in situ for several years within Venezuela, our phylogeographic results indicated YFV movement between Brazil, Trinidad, and Venezuela. Brazil is the major source of YFV introductions into Venezuela, accounting for introductions of Venezuelan strains sampled in 1959, 1961, 1998, and more recently (i.e., independent introductions in 2004 in eastern and western Venezuela; Figure 1). The basal location of a 2004 sequence from the Brazilian Amazon within the western Venezuelan 2004–2007 clade (Figure 1) suggests that the 2005 Venezuelan epizootic/epidemic was initiated by an imported progenitor from the Brazilian Amazon Basin that later evolved independently within Venezuela. Our estimate of the most recent common ancestor for the node containing the 2004–2007 Venezuelan sequences was 2001 (95% highest posterior density 1997–2003), suggesting that the ancestral lineage existed for ≈3 years in Venezuela before its detection in nonhuman primates in 2004.

The 2004 Venezuelan (1A) intermediary descendent between the 1995 and 2009 Trinidad sequences is noteworthy. This cluster of Trinidad isolates was previously used as evidence of enzootic YFV maintenance within Trinidad during interepizootic periods (*4*). Incorporating our new sequences now shows movement between Trinidad and eastern Venezuela. In our phylogeny, strain 1A is the sister lineage of the 2008–09 Trinidad epizootic strains, with the 1995 Trinidadian isolate lying basal to these sequences, with high posterior probabilities (Figure 1). The position of the 2004 Venezuelan 1A sequence possibly reflects importation from Trinidad, which implies that enzootic YFV circulation in Trinidad is not isolated epidemiologically but gave rise to exportation of YFV to Venezuela.

In the absence of more dense sampling, whether bidirectional YFV movement occurs between Venezuela and Trinidad is unclear. Given the proximity and boating traffic between these countries, substantial mixing between their YFV populations would not be surprising. Isolation and sequencing of additional YFV isolates from eastern Venezuela are needed to further evaluate movement between Trinidad and Venezuela.

Complete genomes were sequenced for 5 representative Venezuelan YFV strains from eastern and western Venezuela (Table 1). Comparison of nucleotide and amino acid similarities showed a high degree of conservation across YFV genes (Table 2). The 9 strains selected for comparisons represent the full spectrum of known YFV genetic diversity (Table 2: Figure 2). The most diverse genes shared >97% aa sequence identity, with >99.9% aa sequence identity for most proteins, even though these strains were collected >30 years apart (Table 2). The Bayesian Markov chain Monte Carlo phylogeny based on complete open reading frame sequences strongly supported the inferred maximum clade credibility tree, with all Venezuelan sequences grouping together with strong posterior support. The eastern and western Venezuelan strains grouped separately, with strong support in all of our inferred phylogenies. A total of 5 substitutions delineated the Venezuelan sequences (Figure 2).

**Table 2:** Nucleotide and amino acid divergence among individual YFV genes of 9 representative YFV strains compared to the TVP11767* strain†

Genes	Strain, nucleotide (amino acid) divergence, %								
	BeH622205 (B, 2000)	BeAR513008 (B, 1992)	BeH423602 (B, 1984)	BeH413820 (B, 1983)	BeH394880 (B, 1981)	BeH655417 (B, 2002)	6A (V, 2005)	9A (V, 2007)	10A (V, 2010)
Capsid	98.3 (100)	98.0 (100)	97.2 (100)	91.2 (97.4)	96.3 (100)	97.2 (99.1)	97.2 (99.1)	97.2 (99.1)	96.6 (100)
PreM/M	97.7 (99.4)	96.7 (100)	91.2 (98.1)	97.3 (100)	97.3 (100)	96.2 (99.4)	97.7 (100)	97.7 (100)	97.7 (100)
E	98.2 (100)	96.3 (100)	89.6 (98.6)	96.9 (100)	97.5 (100)	96.6 (100)	97.2 (100)	97.0 (100)	97.6 (100)
NS1	97.9 (99.7)	97.3 (100)	90.5 (99.4)	97.5 (99.7)	98.1 (100)	96.8 (99.7)	98.7 (100)	98.4 (100)	98.6 (100)
NS2A&B	97.4 (100)	96.2 (99.4)	90.4 (99.4)	96.8 (100)	97.6 (100)	95.8 (99.1)	97.2 (100)	96.9 (99.4)	97.6 (100)
NS3	97.4 (100)	96.7 (100)	88.4 (100)	97.4 (100)	97.4 (100)	96.3 (100)	97.4 (100)	97.4 (100)	96.7 (100)
NS4A&B	98.2 (100)	96.8 (99.7)	90.3 (98.7)	97.1 (99.7)	97.8 (100)	97.1 (99.7)	98.1 (99.7)	98.0 (99.7)	98.2 (100)
NS5	98.3 (99.8)	96.6 (99.8)	91.1 (99.1)	96.8 (99.8)	98.0 (100)	96.6 (100)	98.4 (100)	98.1 (100)	98.2 (100)

*First South American complete genome sequence to be published. TVP11767 was isolated in 2009 from an *Alouatta seniculus* specimen in Trinidad.  †YFV, yellow fever virus; B, Brazil; V, Venezuela; M, membrane; E, envelope; NS, nonstructural.

## Conclusions

Our phylogeographic analysis supports in situ evolution of YFV within Venezuela, as well as regionally independent evolution within the country. Brazil was identified as the major source of YFV introductions into Venezuela, and sequence analysis showed that considerable YFV movement may occur between Trinidad and Venezuela. Results of our Bayes factor test for non-zero rates also support the epidemiologic link between Venezuela, Brazil, and Trinidad (Figure 1, panel C). The sequences generated in our study fill a major gap in the geographic sampling of YFV.

Technical Appendix Strains of yellow fever virus used in this study.
