# Supplementary material for: Enzootic Transmission of Yellow Fever Virus, Venezuela
Source: Emerg Infect Dis. 2015 Jan;21(1):99–102. doi: 10.3201/eid2101.140814 (PMC4285243; doi:10.3201/eid2101.140814)
Supplement: Technical Appendix — Strains of yellow fever virus used in this study. [file 14-0814-Techapp-s1.pdf]

Article DOI: http://dx.doi.org/10.3201/eid2101.140814

Enzootic Transmission of Yellow Fever Virus, Venezuela

Technical Appendix

Technical Appendix Table. Strains of yellow fever virus used in this study

GenBank accession no.	Strain name	Origin	Year	Location
AY540433	OBS7937	Santa Cruz	1999	Bolivia
AY540435	OBS8027	Santa Cruz	1999	Bolivia
AY540432	OBS7549	Santa Cruz	1999	Bolivia
AY540431	OBS7687	Santa Cruz	1999	Bolivia
HM582847	FVB0196	Unknown	2006	Bolivia
AY540434	OBS8026	La Paz	1999	Bolivia
AY540468	BeAr527785	Unknown	1994	Brazil
HM582845	BeAr527785	Unknown	1994	Brazil
AY540462	BeAn510268	Unknown	1991	Brazil
AY540451	BeAn232869	Unknown	1973	Brazil
AY540436	BeAr628124	Unknown	2000	Brazil
AY540458	BeH425381	Amapa	1984	Brazil
AY540471	BeH35010	Maranhao	1995	Brazil
AY540456	BeH379501	Maranhao	1980	Brazil
AY540444	BeH107714	Amazonas	1966	Brazil
AY540473	Tennessee	Manaus	1996	Brazil
AY540461	BeH511843	Roraima	1991	Brazil
AY540446	BeH141816	Para	1968	Brazil
AY540460	BeAr511437	Para	1991	Brazil
AY540470	BeAr527547	Para	1994	Brazil
AY540449	BeH203410	Para	1971	Brazil
AY540455	BeH385780	Para	1980	Brazil
AY540454	BeAr350397	Para	1978	Brazil
HM582843	BeH613582	Para	1999	Brazil
AY540459	BeAr424083	Para	1984	Brazil
AY540448	BeAn142027	Para	1968	Brazil
AY540447	BeAr142658	Para	1968	Brazil
AY540445	BeAn142028	Para	1968	Brazil
AY540437	BeH111	Para	1954	Brazil
AY540439	BeAr162	Para	1955	Brazil
AY540438	BeAN131	Para	1955	Brazil
AY540440	BeAr189	Para	1955	Brazil
AY540442	BeAr46299	Para	1962	Brazil
AY540443	BeAr44824	Para	1962	Brazil
AY540441	BeAn23536	Para	1960	Brazil
AY540457	BeH413820	Rondonia	1983	Brazil
AY540472	BeAr544276	Rondonia	1996	Brazil
AY540469	BeAr527198	Minas Gerais	1994	Brazil
HM582848	BeAr631464	Bahia	2001	Brazil
AY540450	BeAr233164	Goias	1973	Brazil
AY540452	BeAr233436	Goias	1973	Brazil
AY540453	BeH233393	Goias	1973	Brazil
HM582849	BeH622205	Goias	2000	Brazil
AY540464	BeAr513008	Mato Grosso do Sul	1992	Brazil
AY540463	BeAr512943	Mato Grosso do Sul	1992	Brazil
AY540465	BeH512772	Mato Grosso do Sul	1992	Brazil
AY540467	BeAr513292	Mato Grosso do Sul	1992	Brazil
AY540466	BeAr513060	Mato Grosso do Sul	1992	Brazil
AY437135	Not available	Unknown	2001	Brazil
AY540476	INS347613	Unknown	1985	Colombia
AY540475	V528A	Unknown	1979	Colombia
AY540474	INS382060	Unknown	2000	Colombia
AY540478	OBS5041	Pastaza	1997	Ecuador
U52398	Ecuador79	Unknown	1979	Ecuador

GenBank accession no.	Strain name	Origin	Year	Location
AY540477	1345	Unknown	1981	Ecuador
AY540480	Jimenez	Unknown	1974	Panama
U52404	Panama74	Unknown	1974	Panama
AY540479	614819	Unknown	1974	Panama
U52410	Peru95	Unknown	1995	Peru
U52409	Peru95	Unknown	1995	Peru
HM582844	FMD1240	Unknown	2007	Peru
HM582846	15094	Unknown	1999	Peru
AY161932	1899/81	Unknown	1981	Peru
AY161943	HEB4245	Junin	1995	Peru
AY161944	HEB4246	Junin	1995	Peru
AY161942	HEB4240	Junin	1995	Peru
AY161937	149	Pasco	1995	Peru
AY161940	OBS2240	Huanuco	1995	Peru
AY161945	OBS2243	Huanuco	1995	Peru
AY161941	OBS2250	Huanuco	1995	Peru
AY161936	HEB4236.153	Pasco	1995	Peru
AY161950	IQT5591	Loreto	1998	Peru
AY161946	ARV0548	S. Martin	1995	Peru
AY161934	ARVO544	S. Martin	1995	Peru
AY161935	HEB4224	S. Martin	1995	Peru
AY161951	OBS7904	S. Martin	1999	Peru
AY161949	OBS6745	Cusco	1998	Peru
AY161947	OBS6530	Cusco	1998	Peru
AY161948	3535098	Cusco	1998	Peru
AY161933	1914	Cusco	1981	Peru
AY161929	1371	Ayacucho	1977	Peru
AY161931	R35740	Ayacucho	1979	Peru
AY161928	1368	Ayacucho	1977	Peru
AY161927	1362/77	Ayacucho	1977	Peru
AY161930	287/78	Ayacucho	1978	Peru
AY161938	Cepa2	Puno	1995	Peru
AY161939	Cepa1	Puno	1995	Peru
U52419	Trinidad79	Unknown	1979	Trinidad
U52416	Trinidad54	Unknown	1954	Trinidad
AY540486	CAREC9515207	Unknown	1995	Trinidad
HM582851	TVP11767	Unknown	2009	Trinidad
HM582840	TVP11649	Unknown	2009	Trinidad
HM582841	TVP11687	Unknown	2009	Trinidad
HM582839	TVP11646	Unknown	2009	Trinidad
HM582842	TVP11640	Unknown	2009	Trinidad
AY540483	CAREC890692	Unknown	1989	Trinidad
AY540485	CAREC891957	Unknown	1989	Trinidad
AY540482	CAREC889920	Unknown	1988	Trinidad
AY540484	CAREC891954	Unknown	1989	Trinidad
AY540481	CAREC797984	Unknown	1979	Trinidad
AY540488	PHO42H	Tachira	1961	Venezuela
AY540487	P128MC	Cojedes	1959	Venezuela
AY540490	35708	Amazonas	1998	Venezuela
AY540489	35720	Amazonas	1998	Venezuela
FJ875515	SPH188002	Unknown	2000	Brazil
FJ875516	SPH188057	Unknown	2000	Brazil
FJ875517	SPH258595	Amazonas	2004	Brazil
FJ875518	SPAn288183	Unknown	2008	Brazil
FJ875519	SPAn288184	Unknown	2008	Brazil
FJ875520	SPAn289568	Unknown	2008	Brazil
HQ123568	PH71191	Unknown	2008	Argentina
HQ123569	PA5342	Unknown	2008	Argentina
HQ123570	PA5343	Unknown	2008	Argentina
HQ123571	PP123	Unknown	2009	Argentina
KM388819	1A	Monagas	2004	Venezuela
KM388817	2A	Guárico	2004	Venezuela
KM388820	3A	Portuguesa	2005	Venezuela
KM388821	4A	Portuguesa	2005	Venezuela
KM388822	5A	Portuguesa	2004	Venezuela
KM388814	6A	Portuguesa	2005	Venezuela
KM388823	7A	Portuguesa	2005	Venezuela
KM388818	8A	Barinas	2006	Venezuela
KM388815	9A	Apure	2007	Venezuela

GenBank accession no.	Strain name	Origin	Year	Location
KM388816	10A	Monagas	2010	Venezuela

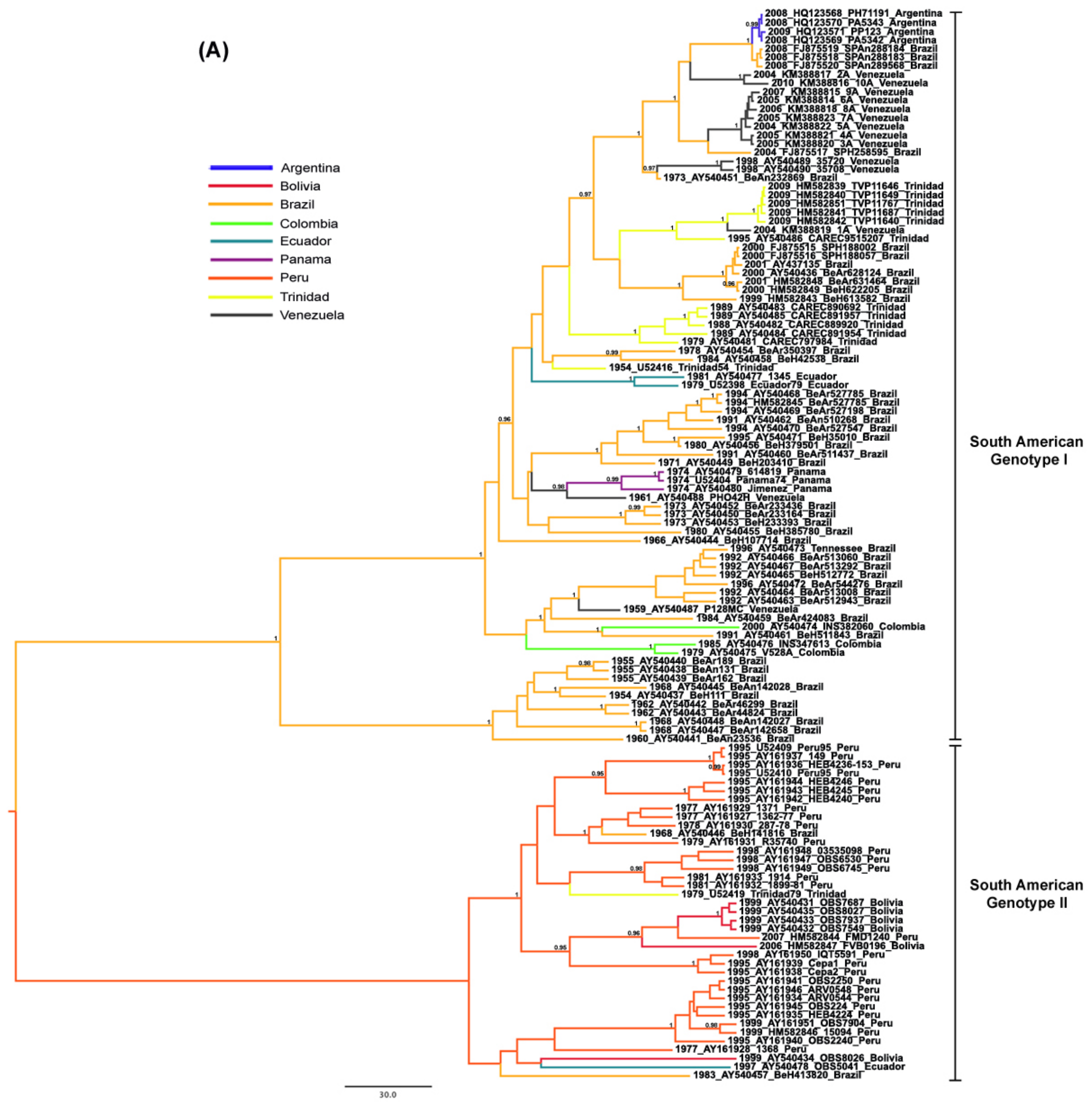

Technical Appendix Figure. Bayesian maximum clade credibility (MCC) tree for YFV in the Americas based on 654 nt of the prM/E fragment. Taxon labels include year of isolation, GenBank accession number, strain designation, and country of isolation. Terminal branches of the tree are colored according to the sampled location of the taxon at the tip. Internal branches

are colored according to the most probable (modal) location of their parental nodes. Nodes with posterior probabilities (clade credibilities) $\geq$0.95 are labeled accordingly in black. Scale bar indicates time in years.
